# Is an oritavancin catheter lock solution active against biofilms of staphylococci and enterococci?

**DOI:** 10.1016/j.heliyon.2025.e41885

**Published:** 2025-01-10

**Authors:** Marta Díaz-Navarro, Emilia Cercenado, Ariadna Monte, Andrés Visedo, Carmen Rodríguez, Ma Jesús Pérez-Granda, Patricia Muñoz, María Guembe

**Affiliations:** a Department of Clinical Microbiology and Infectious Diseases, Hospital General Universitario Gregorio Marañón, Madrid, Spain; b Instituto de Investigación Sanitaria Gregorio Marañón, Madrid, Spain; c School of Medicine, Universidad Complutense de Madrid, Spain; d CIBER Enfermedades Respiratorias-CIBERES (CB06/06/0058), Madrid, Spain; e School of Biology, Universidad Complutense de Madrid, Spain; f Pharmacy Department, Hospital General Universitario Gregorio Marañón, Madrid, Spain

**Keywords:** Oritavancin, Catheter lock solution, Catheter-related bloodstream infection, Activity, Stability

## Abstract

**Background:**

Oritavancin (ORT) is a new single-dose lipoglycopeptide showing *in vitro* activity against staphylococci and vancomycin-resistant enterococci. However, there is no data regarding its potential use as a catheter lock solution are scarce. We constructed an *in vitro* model to analyze the efficacy and stability of an ORT lock solution against the biofilm of staphylococci and enterococci over 7 days at 37 °C.

**Methods:**

We used *Staphylococcus aureus*, *Staphylococcus epidermidis*, and vancomycin-susceptible *Enterococcus faecalis* ATCC strains. We performed a metabolic activity assay using a 2-mg/ml solution of ORT over a 7-day incubation period at 37 °C. The solution was tested against 24-h biofilms of each strain at day 0 and 7. Metabolic activity was measured using the XTT assay, and median absorbance obtained at 490 nm in the spectrophotometer was compared between day 0 and day 7.

**Results:**

The percentage reduction in metabolic activity was 95.3 % between biofilms treated with ORT solution incubated for 7 days and biofilms treated with ORT solution before incubation.

**Conclusion:**

Ours is a proof-of-concept study that shows ORT to be a potential treatment as a catheter lock solution for eradication of staphylococcal and *E*. *faecalis* biofilms. It is needed to further test ORT against more clinical strains and to compare its activity with other antimicrobials in a biofilm model.

## 1 Introduction

Oritavancin (ORT) is an emerging lipoglycopeptide which can act against Gram-positive bacteria by inhibiting bacterial cell wall biosynthesis and by disrupting bacterial membrane [1]. Owing to its pharmacokinetic and pharmacodynamic characteristics, it is used as a single dose and has been demonstrated to be cost-effective [1,2]. In addition, it demonstrated to can act against vancomycin-resistant enterococci (VanA, VanB, and VanC phenotypes) by *in vitro* studies [[3], [4], [5], [6]].

At present, oritavancin is only approved to treat skin and soft tissue infections. However, several studies have demonstrated success rates in other infections, such as deep-seated infections (including those involving prosthetic material), invasive infections and those caused by vancomycin-resistant enterococci (VRE) [[7], [8], [9], [10]].

It is recommended to remove the catheter and administer systemic antimicrobial therapy when there is a catheter-related bloodstream infection (C-RBSI) [11]. However, in long-term difficult-to-replace tunneled catheters, conservative management can be approached as demonstrated in several clinical studies [[12], [13], [14], [15], [16], [17], [18], [19], [20], [21], [22], [23]], even in *Staphylococcus aureus* infections and with hemodialysis tunneled catheter or reservoir ports, in which sometimes systemic therapeutic approaches are different [24].

When conservative management is performed, it is recommended to use antibacterial lock solutions with good anti-biofilm activity in combination with systemic antimicrobial therapy, as it can eradicate bacterial biofilm on catheter surface and it avoids routine catheter exchange [11,19].

Regarding ORT, only one *in vivo* study used the *Enterococcus faecium* VanA strain to create a catheter infection model in rats to which either saline (positive control) or ORT was administered. The authors revealed no infection in peripheral blood in the treated group (0 % *vs.* 75 % of non-treated) [25]. Therefore, there is scarce data on the use of ORT as lock therapy in Gram-positive colonized catheters that cannot be removed.

As it has been demonstrated antibiotic lock solutions can be dwell up to 10 days, the objective of our study was to confirm whether ORT maintained its effectiveness against bacterial biofilms and remained stable in a 2-mg/ml antimicrobial lock solution for 7 days at 37 °C, simulating 7-day catheter lock therapy to Refs. [[26], [27], [28]].

## 2 Methods

We performed an *in vitro* model using the following strains (previously confirmed to be moderate or high biomass producers by the crystal violet binding assay): methicillin-susceptible *S. aureus* (ATCC29213), methicillin-resistant *S. aureus* (ATCC43300), methicillin-resistant *Staphylococcus epidermidis* (ATCC35984), and vancomycin-susceptible *Enterococcus faecalis* (ATCC35186) [29].

The model was based on 24-h mature biofilms in 24-well plates treated with a 2-mg/ml ORT solution.

### 2.1 ORT catheter lock solution

We transferred the 40 ml-reconstituted vial of Tenkasi (10 mg/ml) to 160 ml of glucose saline solution 5 % to finally get a 200-ml ORT solution at a concentration of 2 mg/ml to be administered as a standard catheter lock solution in future clinical settings.

### 2.2 Biofilm procedure

The procedure for biofilm formation followed the description of Peeters et al., with some modifications [30]. Each strain was inoculated in 20 ml of tryptic soy broth (TSB, supplemented with glucose 1 % in *S. epidermidis* strains and enterococci) and incubated in an orbital shaker at 37 °C for 24 h. Then, inoculums were washed in three centrifuge-resuspension cycles with phosphate-buffered saline, and resuspended in 10 ml of TSB to adjust them to 0.5 McFarland turbidity (10^8^ cfu/ml). Finally, 100 μl of the suspension was inoculated in a non–tissue-prepped 96-well plate followed by an incubation of 24 h at 37 °C. Then, plates were washed three times with phosphate-buffered saline followed by a 24-h treatment with 100 μl of lock solution (or saline for positive controls). Analysis was performed using tetrazolium salt (XTT) to assess median (IQR) absorbance values at 490 nm in the spectrophotometer between biofilms treated over a 7-day incubation period at 37 °C and biofilms treated before incubation (day 0).

We calculated percentage reduction of metabolic activity with the following formula:

$$\left[1-\left({\text{abs}}_{\text{treated}\;\text{well}}/{\text{abs}}_{\text{positive}\;\text{control}\;\text{well}}\right)\right]x\;100$$

### 2.3 Statistical analysis

Quantitative variables are expressed as median (IQR). Continuous variables were compared using the *t*-test in the case of non-normally distributed variables (Kruskal-Wallis). All statistical tests were two-tailed. Statistical significance was set at p < 0.05 for all the tests. Statistical analysis was performed using IBM SPSS Statistics for Windows, Version 21.0 (IBM Corp, Armonk, New York, USA).

## 3 Results

**Fig. 1:**
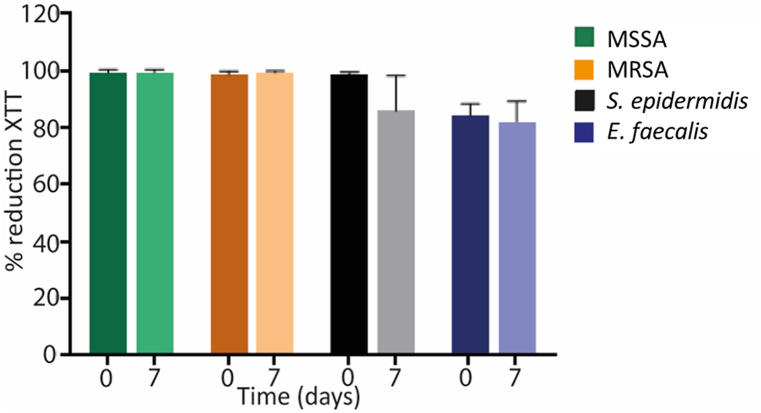
Mean percentage reduction in metabolic activity for each species. **MSSA**, methicillin-susceptible *Staphylococcus aureus*; **MRSA**, methicillin-resistant *Staphylococcus aureus.*

We recorded a 95.3 % reduction in metabolic activity between biofilms treated with ORT solution incubated for 7 days and biofilms treated with ORT solution before incubation. No decrease in the median percentage reduction in metabolic activity was observed for MSSA or MRSA strains after a 7-day incubation time. While the metabolic activity of *S. epidermidis* and *E. faecalis* decreased by a mean of 18.0 % and 8.0 %, respectively, between the study experiments, the difference was not statistically significant (p = 0.513) (Table 1, Fig. 1).

**Table 1:** Median (IQR) percentage reduction in metabolic activity according to days of incubation with oritavancin solution.

Strain	Days of conservation		p
	0	7	
MSSA	100.00 (98.14–100.00)	100.00 (98.29–100.00)	0.796
MRSA	98.51 (97.83–98.51)	99.22 (98.78–99.22)	0.376
*S. epidermidis*	98.82 (97.56–98.82)	80.95 (76.67–80.95)	0.513
*E. faecalis*	86.11 (79.48–86.11)	78.04 (77.78–78.04)	0.513

**MSSA**, methicillin-susceptible *Staphylococcus aureus*; **MRSA**, methicillin-resistant *Staphylococcus aureus.*

## 4 Discussion

Our is a proof-of-concept study showing ORT to be a potential option as a catheter lock solution for eradication of staphylococcal and *E*. *faecalis* biofilms.

ORT has showed successful results in the treatment of deep-seated infections, such as infective endocarditis, C-RBSI, and bone and prosthetic joint infections, despite it is only approved for the treatment of skin and soft tissue infections [9].

Moreover, we observed not only a synergistic *in vitro* effect with other antimicrobials [[31], [32], [33]], but also an *in vivo* effect, as reported by Di Cecco et al., who successfully treated a patient with a vancomycin-resistant *Enterococcus* spp. bloodstream infection with sequential ORT and fosfomycin [34].

Therefore, based on our previous experiments with dalbavancin, in which ORT was successful as a new, alternative catheter lock solution for the management of C-RBSI, we aimed to test ORT in the same scenario [[35], [36], [37]]. ORT not only successfully reduced the rate of metabolic activity against staphylococcal and enterococcal biofilms, but also maintained its efficacy after a 7-day incubation period simulating a 7-day dwell time for catheter lock therapy.

Although our experiment was based on an *in vitro* model, it highlights a promising new application of ORT.

One of the main limitations of our study is that we only tested ORT activity based on indirect measurement of the reduction in metabolic activity compared to a non-treated positive control. Therefore, the correlation with other variables, such as cfu counts or biofilm occupation area quantified by culture and confocal microscopy, respectively, requires further assessment. Another limitation was that we did not include an *E. faecium* strain (both vancomycin-resistant and -susceptible). We had collected an ATCC strain of vancomycin-susceptible *Enterococcus faecium*, although we were unable to create a stable biofilm for it and one that was robust enough to analyze the reduction in metabolic activity after treatment with ORT. Therefore, future studies are needed to test ORT against a large collection of clinical strains and to compare its activity with other antimicrobials in a biofilm model.

## 5 Conclusions

Our preliminary *in vitro* results suggest that ORT is a suitable catheter lock solution for the conservative treatment of C-RBSI caused by staphylococci and *E. faecalis*.

## CRediT authorship contribution statement

**Marta Díaz-Navarro:** Writing – original draft, Software, Methodology, Formal analysis, Conceptualization. **Emilia Cercenado:** Writing – original draft, Methodology. **Ariadna Monte:** Methodology. **Andrés Visedo:** Methodology. **Carmen Rodríguez:** Writing – original draft, Methodology. **Ma Jesús Pérez-Granda:** Writing – original draft, Methodology. **Patricia Muñoz:** Writing – review & editing, Validation, Supervision. **María Guembe:** Writing – review & editing, Visualization, Supervision, Resources, Funding acquisition, Conceptualization.

## Consent for publication

Not applicable.

## Declarations/ethics

Not applicable, as it was an *in vitro* study not including clinical strains nor patients’ data.

## Data availability statement

The datasets used and/or analyzed during the current study are available from the corresponding author on reasonable request.

## Funding source

MG was supported by the Miguel Servet Program (ISCIIIMICINN, MSII18/00008) from the Health Research Fund (FIS) of the Carlos III Health Institute (ISCIII), Madrid, Spain. MD-N was supported by the FIS of the ISCIII (FI22/00022). AV was supported by the Consejería de Educación, Juventud y Deporte de la Comunidad de Madrid and Fondo Social Europeo (PEJD-2021-TL/BMD-21113). The study was partially funded by grants from the Fundación Mutua Madrileña (FMM21/01), ISCIII (PI21/00344), IiSGM (2022-PI-II-COOPTR-01) and the European Regional Development Fund (FEDER) “A way of making Europe.”

## Declaration of competing interest

The authors declare the following financial interests/personal relationships which may be considered as potential competing interests:Maria Guembe reports financial support was provided by 10.13039/501100004587Carlos III Health Institute. Marta Diaz-Navarro reports financial support was provided by 10.13039/501100004587Carlos III Health Institute. Andres Visedo reports financial support was provided by 10.13039/100012818Community of Madrid Department of Education Science and Universities. Maria Guembe reports financial support was provided by Mutua Madrileña Foundation. Maria Guembe reports financial support was provided by Gregorio Maranon Health Research Institute. If there are other authors, they declare that they have no known competing financial interests or personal relationships that could have appeared to influence the work reported in this paper.
